# The Hospital World

**Published:** 1911-05-13

**Authors:** 


### The Hospital World.

### "Marshall's Gatherings" at St. George's.

The late medical superintendent of St. George's Hospital, whose death last month was recorded in our issue of April 8, is the subject of an appreciation in the May number of his hospital's *Gazette*. There one who was a frequenter of "Marshall's Room" at the Cottage describes the well-known Saturday afternoons as follows: "Marshall made his quarters practically a common room for the members of the Cottage. But it was more than this, for it was a common room of which were free any former 'Cottagers,' as he loved to call them, who happened to be in the neighbourhood, whether practising or on a chance visit, and also any member of the staff of the hospital who cared to drop in. Thus it was that we juniors met over the card table, or chatting and smoking on his sofa and armchair, men whose names we knew and respected; and thus it was that more than one career was turned into its proper direction and more than one prosperous partnership initiated through a common friendship with Marshall. Saturday afternoon was the special time for Marshall's gatherings, for on that day he was tied to the hospital premises, and on that afternoon even those who were 'in-weeking' generally got a spare hour or so, while his friends from outside knew that on Saturdays they could count on finding at least one or two congenial spirits. In the winter time on such afternoons there would be two, or often three, tables hard at 'work.' Meanwhile, very likely your friend N———, whose labours in the wards for the day were over, played on the piano selections from those innumerable old operas and operettas, the scores of which, piled beside the piano, were an inexhaustible storehouse of joy to Marshall. He loved to hear the old airs which had been the gaiety of his younger days. In these matters he was a thorough conservative." The same writer recalls the unpopularity of Mr. Marshall's appointment, due to the fact that the post was a new creation, which only his own tact and charm succeeded in reconciling to the residents of the seventies. He retired in 1905, after thirty years' service.

### The New Member of the Medical Council.

It was notified in a *Gazette* of last week that the King has been pleased to nominate Sir Francis Henry Champneys, Bart., M.D., to be, for the term of five years from May 23, 1911, a member of the General Council of Medical Education and Registration of the United Kingdom. It will be remembered that Sir Francis is now Chairman of the Central Midwives Board.

### A Pioneer President at Derby.

The Derbyshire Royal Infirmary has lost one of its best friends in the person of the late Sir Henry Howe Bemrose, who died shortly before midnight on Thursday last week. Sir Henry was a man of most striking personality, and was prominently connected with the religious, political, and philanthropic life of Derby, his native town. Born in November 1827, he was the eldest son of the late Mr. William Bemrose, who founded the great printing business which bears his name. He was educated at Derby Grammar School, and at King William's College, Isle of Man. Sir Henry took a very large part in the municipal work of the Borough, and was unanimously elected Mayor in November 1877. He was Member of Parliament for the Borough in 1895, defeating on that occasion the late Sir William Harcourt, the then Chancellor of the Exchequer. When the proposals for the building of the new Infirmary were placed before the public, Sir Henry very strongly urged the necessity for carrying out the schemes proposed by the Governors. Sir Henry was a member of the Building Committee, and was a generous contributor to the Building Fund. In 1897 he was elected President of the Infirmary, and during that year the manner of the keeping of the accounts was changed, and the uniform system of accounts adopted. During his presidency a large sum was obtained towards the Building Fund, and the Susan Evans Wing, erected by Mr. Walter Evans, in memory of his first wife, was opened. At the anniversary services held during his year of office Sir Henry succeeded in securing Archbishop Temple to preach at the morning service, and Dean Farrar to preach the sermon in the evening. Sir Henry has been the Honorary Secretary to the Infirmary Saturday Committee since its inception, and was also Chairman of the Midland Institute for the Deaf and Dumb.

A vote of condolence has been passed by the Board, which summarises his services to the hospital.

### The Hayter Memorial at Fulham.

The memorial to the late Mr. Owen Edward Hayter, former Chairman of the House Committee of the Cancer Hospital, Fulham Road, was publicly set up on Tuesday. It took the form of a stained-glass window in the chapel, which was unveiled and dedicated by the Bishop of London. A very distinguished company was present at the service, including the Earl of Northbrook, President of the Hospital, Lord Ludlow, Viscount Mountmorres, Lord Haversham, and Mrs. Owen Hayter and Mr. Cecil Hayter, which was conducted by Mr. Farington Dounes, the hospital chaplain, assisted by Archdeacon Wilberforce. The Bishop delivered a fitting address, for he has often testified to the value of personal service in hospital life.

### The Queen and a Haslar Patient.

Mr. George de Matose, the third-class naval writer who attracted her Majesty's notice when she visited Haslar Hospital last July, has died of carcinoma of the lungs. It will be remembered that de Matose was in hospital as the result of injuries suffered in a Service football match. His right leg eventually had to be amputated, and after leaving the hospital he wrote to her Majesty explaining that he was without employment and dependent on his mother, a widow. The Queen's intervention secured him a post as a writer in Portsmouth Dockyard, but in February last his lungs were attacked. Her Majesty was again informed of his condition, and sent a recognised authority to see him, who was obliged to admit the case a hopeless one. A sad feature of the case was the young man's impression that if only he could be admitted to Haslar Hospital he would recover there.

### ELECTIONS AND APPOINTMENTS.

The retirement of Mr. J. R. Wheldon from the office of secretary to the Ingham Infirmary, South Shields, has led to the appointment of a whole-time secretary in the person of Mr. John Potter.

Mr. Francis J. A. Wood and Mr. Shrewsbury Smith have been elected respectively chairman and vice-chairman of the Worcester General Infirmary for the ensuing year.

